# Chinese Minimally Invasive Percutaneous Nephrolithotomy for Intrarenal Stones in Patients with Solitary Kidney: A Single-Center Experience

**DOI:** 10.1371/journal.pone.0040577

**Published:** 2012-07-10

**Authors:** Zhichao Huang, Fajun Fu, Zhaohui Zhong, Lei Zhang, Ran Xu, Xiaokun Zhao

**Affiliations:** 1 Department of Urology, Second Xiangya Hospital, Central South University, Changsha, Hunan, China; 2 Department of Urology, Changsha Central Hospital, Changsha, Hunan, China; University of Sao Paulo Medical School, Brazil

## Abstract

**Objective:**

To report our experience with Chinese minimally invasive percutaneous nephrolithotomy (Chinese MPCNL) to manage patients with intrarenal stones in solitary kidney, and evaluate the safety, efficiency and feasibility of this technique.

**Methods:**

Forty-one patients with intrarenal stones in solitary kidney underwent Chinese MPCNL in our department from March 2009 to February 2011. Demographic characteristics, operative parameters, number of tracts, stone-free rates (SFRs), stone analyses, hemoglobin levels, nephrostomy tube removal time, hospitalization time, and complications were evaluated. Serum creatinine (Scr) and glomerular filtration rate (GFR) were measured preoperatively, postoperatively at 1 month, and each follow-up visit. The 5-stage classification of chronic kidney disease (CKD) was used according to the National Kidney Foundation guidelines.

**Results:**

The initial stone-free status was achieved in 35 (85.4%) patients after Chinese MPCNL. The mean follow-up time was 16.9±4.7 months (range: 12–24), and the final SFR improved to 97.6% after auxiliary procedures. Among all patients, complex stones were detected in 26 (63.4%) patients, and 9 (22.0%) required multiple tracts. The mean operative time and mean hospitalization time were 71.3±23.5 min (range: 40–139) and 6.1±0.5 days (range: 5–11), respectively. During preoperative period and postoperative period (1 month), Scr were 132.1±41.3 umol/L (range: 78.2–231.4) and 108.9±30.7 umol/L (range: 71.6–136.9), respectively (P<0.05), while GFR were 74.9±24.2 ml/min (range: 35–110) and 83.9±27.4 ml/min (range: 65–110), respectively (P<0.05). According to CKD classification, the renal function was stable, improved, and worse in 29 (70.7%), 11 (26.8%), and 1 (2.5%) patients, compared with the preoperative levels. No patient progressed to end-stage renal disease requiring dialysis.

**Conclusions:**

Our experience with Chinese MPCNL demonstrates that it is safe, feasible and efficient for managing the intrarenal calculi in solitary kidney with a low complication rate. At long-term follow-up, renal function stabilized or even improved in the majority of patients with solitary kidney.

## Introduction

Percutaneous nephrolithotomy (PCNL) has become the first method of choice for patients with large and complex calculi since its introduction in 1976 [1]. The 2005 American Urological Association (AUA) Nephrolithiasis Clinical Guidelines recommend PCNL as the first-line treatment for calculi greater than 500 mm^2^ [2]. The excellent stone-free rate (SFR) following PCNL is 78% to 95% [3]. However, PCNL can still be associated with significant complications, such as uncontrolled hemorrhage, injury to collecting system and surrounding viscera, urinary leakage, sepsis, loss of kidney, or even death [3]. Therefore, PCNL poses a significant risk, especially for patients with solitary kidney. Meanwhile, the standard size tract (26–30 F) may be too large for pediatric kidneys and some undilated adult kidneys. To decrease morbidity, especially uncontrolled hemorrhage, some urologists have modified the technique of standard PCNL by performing it with a miniature endoscope by way of a small size tract (12–20 F) and named it as MPCNL [4], [5], [6]. Although MPCNL has exhibited advantages with respect to hemorrhage, injury to renal parenchyma, postoperative pain, and shortened hospitalization time [4], [7], [8], the disadvantages of need for specialized equipments and relatively low efficiency to fragment large stones than standard PCNL limited its indications. The indications of MPCNL are only limited to pediatric patients or adult patients with stone diameter less than 2 cm or as a secondary tract of standard PCNL [4], [5], [6].

In China, urologists have modified PCNL technique since the 1990s, using an 8/9.8 F rigid ureteroscope via the 14–18 F percutaneous tract provided by fascial dilator and matched peel-away sheath to manage the upper urinary tract calculi and termed it as Chinese MPCNL [9], [10].

In this study, we evaluated the safety, efficiency and feasibility of Chinese MPCNL on management of intrarenal stones in solitary kidney and evaluated the long-term renal function. To our knowledge, few studies have been performed.

## Materials and Methods

We obtained approval for this study from the ethics committee of our hospital. Meanwhile, we obtained informed consent from all participants in our study. The informed consent was written and specified in the operative consent. Between March 2009 and February 2011, 41 patients with intrarenal stones in solitary kidney (congenital in 4 patients, 9.8%; contralateral nephrectomy in 30 patients, 73.2%; and nonperfused kidney in 7 patients, 17.0%) underwent Chinese MPCNL in our department. Patient demographic characteristics, such as age, gender, previous renal intervention history were studied. Patients were evaluated preoperatively with blood routine tests, coagulation tests, urinalyses, urine cultures, and serum biochemistry. Before operation, computed tomography (CT) was performed routinely to assess the number, location, and size of the stone, identify the collecting system anatomy, and provide an anatomical proof for establishing percutaneous tract. Prophylactic preoperative wide-spectrum antibiotics were administered to patients with positive urine culture result according to the antibiotic susceptibility tests. Stone size was evaluated as the surface area and calculated according to the European Association of Urology (EAU) Guidelines [11]. Stones were classified as simple (isolated caliceal or renal pelvis stones) or complex (renal pelvis stones accompanying caliceal stones, complete or partial staghorn stones), regardless of its size.

A plain X-ray of the kidneys, ureters, and bladder (KUB) was performed 24 to 48 hours after operation to assess the effect of surgery and identify the position of D–J stent. In patients with complete stone-free or clinically insignificant residual fragments (CIRFs), the nephrostomy tube was clamped when the drainage was clear and subsequently removed. The D–J stent would be removed 2 to 3 weeks later. Otherwise, the flexible ureteroscopy and extracorporeal shock wave lithotripsy (ESWL) were performed as auxiliary procedures. All patients were assessed by CT one month after the final procedure to confirm the final SFR. Complete stone-free was defined as the absence of any fragments in kidney or had CIRFs, defined as ≤4 mm, nonsymptomatic, nonobstructive and noninfectious residual fragments [12].

Scr was measured before the operation, on the first postoperative day, and at each follow-up visit. GFR was determined preoperatively, postoperatively at 1 month and each follow-up visit. Calculated GFR was determined using the Cockroft and Gault formula, GFR = [(140-age)(weight in Kg)(0.85 for women)]/72×Cr (mg/100 ml) [13]. The CKD stages 1–5 were stratified as either normal, mild, moderately, severely decreased GFR, or those requiring dialysis or a kidney transplant (>90, 60–89, 30–59, 15–29, and <15 ml/min) according to the National Kidney Foundation guidelines [14]. The Scr, GFR, and CKD stage during preoperative period were compared with those at the follow-up visit.

All patients were followed for at least 12 months, especially patients with residual fragments. The first follow-up visit was performed 1 month after the final procedure and then patients were followed every 3 months during the first year and every 6 months thereafter. At each follow-up visit, urinalysis, urine culture, serum biochemistry, blood routine test, KUB+ intravenous urography (IVU), and renal ultrasound were performed to confirm the presence of urinary tract infection (UTI), hydronephrosis, fragments growth and stone recurrence.

Statistical analysis was performed using SPSS 17.0 (SPSS, Inc., Chicago, IL). The continuous variables were compared with student’s t tests and Wilcoxon tests. Differences resulting in a P-value of <0.05 were considered significant.

### Surgical Technique

**Figure 1:**
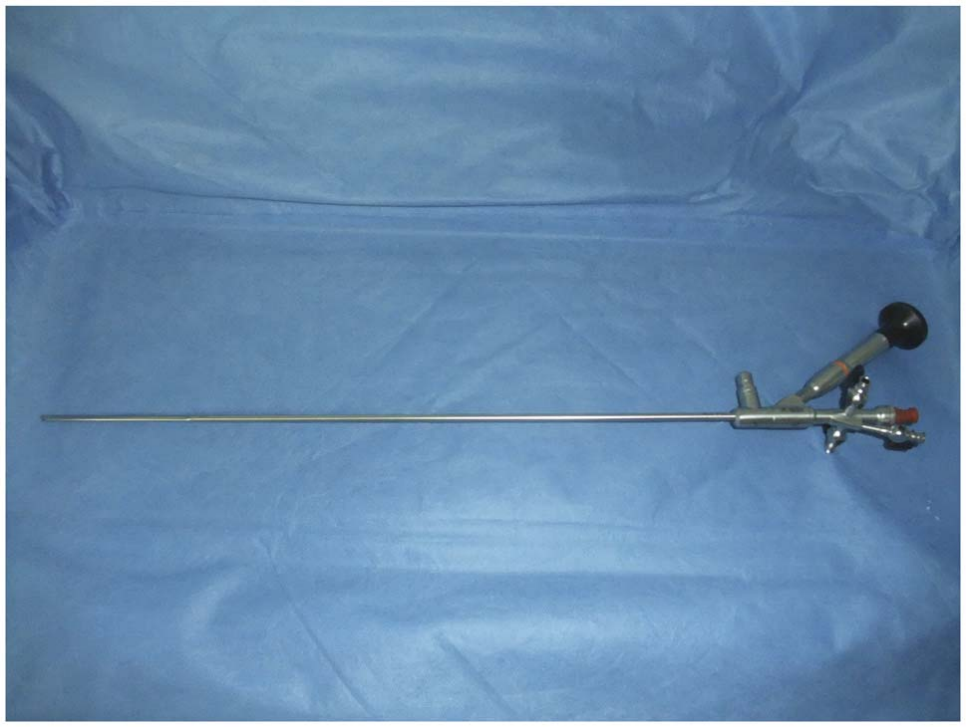
The 8/9.8 **F rigid ureteroscope (Richard Wolf, German).**

All procedures were performed under continual epidural anesthesia or general anesthesia. Patient was firstly placed in a split lower limbs position, and a 5 F ureteral catheter was inserted into the target ureter under direct ureteroscopic vision. Then, the patient was turned into a prone position with a pack under the abdomen to minimize lumber lordosis. Access to the designed calyx was performed by the urologists with the help of fluoroscopy or ultrasonography using an 18-gauge needle (Cook Urological, Spencer, IN). A posterior middle calyx puncture via the 11^th^ intercostal space between the posterior axillary line and scapula line was preferred. Tract dilation was serially performed using fascial dilator (Cook Urological, Spencer, IN) from 8 F to 18 F, and a matched peel-away sheath was placed. The stones were fragmented with a holmium laser or pneumatic lithotripter through an 8/9.8 F rigid ureteroscope (Richard Wolf, German) (Figure 1). The big fragments were removed with a forceps, while small fragments (<0.3 cm) were pushed out with an endoscopic pulsed perfusion pump. Flexible ureteroscopy was used to decrease the necessity of multiple tracts and determine the stone-free status. Finally, a 5 F D–J stent (Cook Urological, Spencer, IN) was inserted via the percutaneous tract with the assistance of guidewire, and a matched size nephrostomy tube was inserted in the collecting system. The operative time was calculated from the time of percutaneous puncture to the completion of nephrostomy tube placement.

## Results

Our study identified 41 patients, including 27 (65.9%) men and 14 (34.1%) women. Patients’ age was 16 to 69 years (mean: 51.4±14.7). Mean stone size was 912±517 mm^2^ (range: 300–1800). Preoperative urine cultures were positive in 7 (17.1%) patients, including 4 patients with *E. coli*, 2 with *Pseudomonas aeruginosa* and 1 with *Enterococcus faecalis*. All these infections were treated with culture-specific antibiotics. Six (14.6%) patients had a previous renal intervention history in the same kidney (1 previous open surgery; 2 previous ESWL; 3 previous PCNL). Thirty-two (78.0%) patients managed with a single tract, and 9 (22.0%) required multiple tracts. The mean operative time was 71.3±23.5 min (range: 40–139). The initial SFR was 85.4% after the procedure of Chinese MPCNL, and 6 (14.6%) had significant residual calculi. Of these patients, four (9.8%) were performed with ESWL, and 2 (4.9%) needed a flexible ureteroscopy procedure. After all auxiliary procedures, forty (97.6%) patients achieved the stone-free status. Finally, there were 2 (4.9%) patients with CIRFs and one with significant residual calculi (7 mm). All of these 3 patients were followed up, and the size of the fragments was stable. During the long-term follow-up, spontaneous stone passage was noticed in 1 of 2 patients with CIRFs. The patient, stone and operative characteristics are shown in Table 1.

**Table 1:** Patient, stone demographics and operative characteristics.

Age (Mean±SD, range) (year)	51.4±14.7 (16–69)
Gender (n, %)	
Male	27 (65.9%)
Female	14 (34.1%)
Cause of solitary kidney (n, %)	
Congential	4 (9.8%)
Previous nephrectomy	30 (73.2%)
Nonperfused kidney	7 (17.0%)
Stone side (n, %)	
Left	19 (46.3%)
Right	22 (53.7%)
Stone number (n, %)	
Single	30 (73.2%)
Multiple	11 (26.8%)
Stone type (n, %)	
Simple	15 (36.6%)
Complex	26 (63.4%)
Stone size (Mean±SD, range) (mm^2^)	912±517 (300–1800)
Previous renal intervention history (n, %)	
Open surgery	1 (2.4%)
ESWL	2 (4.9%)
PCNL	3 (7.3%)
Grade of hydronephrosis (n, %)	
None or mild	13 (31.7%)
Moderate or severe	28 (68.3%)
Positive preoperative urine culture (n, %)	7 (17.1%)
Preoperative serum hemoglobin (Mean±SD, range) (g/L)	114.7±26.9 (87–141)
Preoperative serum creatinine (Mean±SD, range) (umol/L)	132.1±41.3 (78.2–231.4)
Baseline GFR (Mean±SD, range) (ml/min)	74.9±24.2 (35.0–110.0)
Number of tract (n, %)	
Single	32 (78.0%)
Multiple	9 (22.0%)
Operative time (Mean±SD, range) (min)	71.3±23.5 (40–139)
Stone-free rate (n, %)	
Initial stone-free rate	35 (85.4%)
Final stone-free rate	40 (97.6%)
Auxiliary procedure requirement (n, %)	
ESWL	4 (9.8%)
Flexible ureteroscopy	2 (4.9%)
Nephrostomy tube removal time (Mean±SD, range) (day)	4.4±0.3 (4–7)
Hospitalization time (Mean±SD, range) (day)	6.1±0.5 (5–11)
Stone composition (n, %)	
Calcium oxalate	16 (39.0%)
Struvite	7 (17.1%)
Cystine	1 (2.4%)
Uric acid	2 (4.9%)
Mix	4 (9.8%)
Calcium oxalate and phosphate	11 (26.8%)

ESWL: extracorporeal shock wave lithotripsy.

PCNL: percutaneous nephrolithotomy.

GFR: glomerular filtration rate.

SD: standard deviation.

The mean Scr were 132.1±41.3 umol/L (range: 78.2–231.4), 108.9±40.7 umol/L (range: 71.6–136.9), and 107.1±35.6 umol/L (range: 71.0–137.1) during the preoperative period, one month follow-up, and at the last follow-up visit (≥12 months). A statistical significance was detected in the one month follow-up period Scr when compared to the preoperative Scr (P<0.05). The same results were detected in GFR before operation it was 74.9±24.2 ml/min (range: 35.0–110.0) and by the end of one month follow-up it was 83.9±27.4 ml/min (range: 65.0–110.0) with statistical significance (P<0.05). However, our results showed no statistically significant difference both Scr and GFR from one month to the last follow-up visit. During the preoperative period, 11 (26.8%) patients had CKD stage 1, 15 (36.6%) had stage 2, 12 (29.3%) had stage 3, and 3 (7.3%) had stage 4, while CKD stage revealed stable, improved and worsening diseases in 29 (70.7%), 11 (26.8%) and 1 (2.5%) of patients at the last follow-up visit. During the whole follow-up period, no patient progressed to end-stage kidney disease requiring dialysis.

There were no intraoperative complications in all cases. Postoperative complications occurred in 5 (12.2%) patients. The details of complications are presented in Table 2. Five patients had postoperative fever (temperature of 38.5°C or greater), which was resolved spontaneously in one, while the remaining four patients (9.8%) with preoperative UTIs (*E. coli* in 2 patients, *Pseudomonas aeruginosa* in 1 patient and *Enterococcus faecalis* in 1 patient) were treated with a complete culture-specific antibiotics until body temperature, urinalysis and urine culture were normal. The stone composition in these 4 patients was struvite (3/4, 75%) and calcium oxalate (1/4, 25%). During the long term follow-up, the renal function remained stable in 3 of 4 patients. Only one patient had a decrease in renal function, but no one progressed to the end-stage kidney disease requiring dialysis. All postoperative urine culture results were coincident with the preoperative results. Although all preoperative UTIs were treated with culture-specific antibiotics, the UTIs were not eliminated thoroughly without removing the infectious stones in some cases. Four (9.8%) patients had hemorrhage, and only one required blood transfusion when the hemoglobin level dropped below 80 g/L. All patients with hemorrhage were cured conservatively, and no one received angioembolization. Two patients had prolonged hospitalization time because of urinary leakage from nephrostomy tract after removal of the tube, which was resolved spontaneously without any specific intervention. No sepsis and other complications were detected in any patients after interventions.

**Table 2:** Details of complications.

Postoperative fever	5 (12.2%)
Urinary tract infection	4 (9.8%)
Hemorrhage and hematuria	4 (9.8%)
Blood transfusion	1 (2.4%)
Prolonged tract leakage	2 (4.9%)

## Discussion

Treatment of patients with solitary kidney having intrarenal calculi is one of the most challenging problems in urology. An untreated intrarenal stone is likely to destroy the renal function and/or cause life-threatening sepsis [15]. The optimal goal of surgery is complete removal of the stone to prevent further stone formation and any correlative infection, and to preserve the renal function as far as possible [2]. For intrarenal stone, there are several alternative treatments, such as ESWL, PCNL, combination therapy, and open surgery [2], [16], [17]. Because of the relatively low SFR in managing large stone, risk of steinstrasse, and a high rate of retreatment, ESWL monotherapy has a very limited indication [17]. According to the AUA Guidelines, PCNL is characterized with a SFR of 74 to 83%, a blood transfusion rate of 14 to 24%, an acute complication rate of 15%, an auxiliary treatment rate of 18%, and has many advantages when compared with treatment alternatives such as ESWL monotherapy, combination therapy, and open surgery, especially in managing the complex or staghorn calculi [18]. Although standard PCNL is a well-recognized safe and efficient treatment for intrarenal calculi, it can still be associated with significant complications, such as uncontrolled hemorrhage, sepsis, injury to collecting system and surrounding viscera, even loss of kidney. The blood transfusion rates have been reported from 0.8% to 45% [19]. El-Nahas et al [20] demonstrated that the presence of solitary kidney was a significant risk factor of hemorrhage because compensatory hypertrophy of the renal parenchyma is a physiological response to solitary kidney. It is more likely to increase the risk of hemorrhage when urologists puncture and dilate the thick renal parenchyma because of damaging more renal tissue and blood vessel. Uncontrolled hemorrhage may require angioembolization or even nephrectomy [21]. Furthermore, major complications can lead to significant mortality, especially in patients with solitary kidney. Meanwhile, in clinical practice, the standard tract and endoscope may be too large for managing pediatric kidneys and some undilated adult kidneys.

To decrease the invasiveness and broaden the indication, Chinese urologists have modified the technique by using an 8/9.8 F rigid ureteroscope to manage the upper urinary tract calculi via 14–18 F percutaneous tract provided by fascial dilator and matched peer-away sheath. Compared with the tract of standard PCNL (26–30 F), the small tract (14–18 F) of Chinese MPCNL obviously reduced the damage of renal parenchyma and vessel. During the small tract dilation, the fascial dilator was pushed ahead with rotation, which pushed the vessels aside without injury. In our study, there was only one patient of hemorrhage that needed blood transfusion, and no uncontrolled hemorrhage was detected. The patient who needed blood transfusion, when the hemoglobin dropped below 80 g/L, had a complete staghorn stone with preoperative UTI (*E. coli*) and was treated conservatively with blood transfusion, hemostatic agents, bed rest and clamping the nephrostomy tube, which was resolved spontaneously without a super-selective embolization. The rates of transfusion and uncontrolled hemorrhage were significantly lower than those of standard PCNL previously reported in literature [22]. Meanwhile, approaching the collecting system through the posterior middle calyx provided the most direct and shortest tract from skin to collecting system, and may enable to access the majority of the collecting system even proximal ureter via the small endoscope with minimal deformity and torque on both the renal parenchyma and endoscope to manage the complex or staghorn stone through single tract [23]. Torquing the endoscope against the renal parenchyma to access calyx is one of the most important reasons of hemorrhage and has a positive correlation with high rates of blood transfusion and urinary leakage [24]. Taking all these factors into consideration, we believe that Chinese MPCNL has less invasiveness than standard PCNL. To shorten the operative time, small fragments were flushed out by forceful pulse stream produced by a pulse perfusion pump, and the big fragments were removed by forceps under direct vision of endoscope. Many researchers claimed that the small tract would obviously increase the intrapelvic pressure and result in backflow of irrigation fluid containing endotoxin or bacteria which would induce to bacteremia or even sepsis. Zhong W et al reported the mean intrapelvic pressures were 24.55, 16.49, 11.22, and 6.64 mmHg with the 14 F, 16 F, 18 F, and double-16 F tracts, which remained lower than the level causing backflow (30 mmHg) during Chinese MPCNL [8]. The low intrapelvic pressure is associated with the seldom possibility of postoperative fever and sepsis. In patients with staghorn or complex stones, it was usually hard to achieve complete stone-free with a single tract, especially when the residual calculi located in a calyx parallel to the previous tract. Combined ESWL and flexible ureteroscopy can reduce the need of multiple tracts to manage complex or staghorn calculi. Furthermore, with the advancements in flexible ureteroscopic technology, the need of combined ESWL has been decreased [25]. Combined ESWL is considered for patients with small residual stone (≤2 cm) whose general medical condition prohibits the use of multiple tracts or flexible ureteroscopy. The combined PCNL and flexible ureteroscopy can effectively reduce the need of multiple tracts in complex or staghorn stones with a low rate of complication and discomfort, but does not significantly impact the final SFR and operative time [26]. For patients with a large residual stone burden, the multiple tracts Chinese MPCNL should be considered, because it not only decreased the intrapelvic pressure, but also flushed out the small fragments from the other tract, which obviously shortened the operative time and increased the SFR without increasing the potential morbidity [23]. In our opinion, Chinses MPCNL can broaden its indication for all kinds of upper urinary tract calculi that need standard PCNL intervention [27].

Controversy remains on the issue of whether Chinese MPCNL has the potential effect on renal function of solitary kidney. Traxer et al [28] investigated the extent of renal parenchyma injury in pigs underwent percutaneous puncture. They demonstrated a mean estimated parenchyma scar volume of the 30 F tract was 0.40 ml, which was equal to a mean parenchyma loss of 0.91%. Compared to the overall renal volume, the parenchyma scar resulting from the percutaneous tract is very small, so the influence of renal function induced by percutaneous tract can be ignored. Streem et al [29] reported Scr had no difference at one month in 5 patients who underwent PCNL with solitary kidney. Canes et al [30] reported that the renal function was preserved or even slightly improved, and the GFR level significantly increased one year after PCNL. In our study, a significant improvement in Scr and GFR was detected from preoperative period to one month follow-up. Mean Scr before Chinese MPCNL was 132.1±41.3 umol/L (range: 78.2–231.4) compared to 108.9±30.7 umol/L (range: 71.6–136.9) by the end of the one month follow-up period. The same results were observed in GFR. The mean preoperative GFR was 74.9±24.2 ml/min (range: 35.0–110.0) and calculated at 83.9±27.4 ml/min (range: 65.0–110.0) during the one month follow-up period. However, during the long-term follow-up, the renal function of some patients may gradually aggravate. The comorbidities in these patients such as hypertension, atherosclerosis, diabetes mellitus, and other diseases that could damage the kidney were the risk factors for worsening the renal function. In our study, one patient with decreasing renal function at the long term follow-up was diabetic.

Previous study has demonstrated that the patients with diabetes mellitus, large stone burden, UTI and impaired renal function (IRF) were more likely to require longer hospitalization time [31], [32]. Patients with diabetes mellitus have a significantly higher incidence of perioperative complications, including uncontrolled hemorrhage requiring blood transfusion and severe UTI causing life-threatening sepsis. Meanwhile, the relationship between the hospitalization time and stone burden may be explained by longer operative time, higher complication rates of hemorrhage or urine extravasation, and increased requirements for auxiliary procedures in patients with a large stone burden. Furthermore, patients with IRF are usually anemic, and have coagulopathy. Therefore, the incidence of uncontrolled hemorrhage requiring blood transfusion or even super-selective embolization is higher in these patients. In our study, the mean hospitalization time was 6.1±0.5 days (range: 5–11 days), which was longer than previously reported by other authors. Mishra S et al prospectively compared 26 patients with MPCNL and 26 with standard PCNL. The results demonstrated that significant advantages of MPCNL over standard PCNL in terms of reduced hospitalization time (3.2±0.8 vs 4.8±0.6 days, P≤0.001) [33]. Akman T et al retrospectively reported on 47 patients underwent PCNL with a solitary kidney. The mean hospitalization time was 2.87±1.57 days [22]. China is a developing country, so Chinese MPCNL has been applied just in some major clinic. Because of the immature community medical system, patients are unwilling to discharge unless they can make a full recovery. What’s more important, all preoperative examinations and preparations must be performed in the hospitalization according to the medical insurance system, which spends almost an extra 3 days and artificially increases the hospitalization time. Similarly, Zhong W et al reported on 29 Chinese patients with staghorn calculi ranging from 8.8 to 22.8 cm^2^ (mean 11.7 cm^2^) underwent Chinese MPCNL. Mean hospital stay was 9.8 days (range: 6–13 days) [34]. These reasons may explain why the hospitalization time in our study was longer than previously reported by other authors.

Recent interest in tubeless PCNL, in which a D–J stent is used in place of the nephrostomy tube, results from hopes of a shorter hospitalization time and a decrease in the discomfort associated with standard PCNL. Several studies have demonstrated that the use of tubeless procedure presents a shorter hospitalization time. Akman T et al retrospectively reviewed 1658 patients with renal calculi underwent PCNL. The mean hospitalization time was 2.89±1.66 days (range: 1–21 days). Furthermore, the mean hospitalization time was 2.93±1.70 days and 2.12±0.53 days in patients underwent the standard PCNL and tubeless PCNL procedures, respectively (P<0.0001). According to their outcome of multivariate analysis, the tubeless procedure was variables diminishing the hospitalization time (P = 0.0001, OR = 0.23) [31]. Borges CF et al performed a systematic review with meta-analysis to compare tubeless versus conventional PCNL. Meta-analysis of data showed a benefit of shorter hospitalization time in tubeless PCNL group than conventional group (Mean difference: −1.11; CI 95% = −1.55 to −0.68; P<0.00001) [35]. However, tubeless PCNL was indicated in patients with mild-to-moderate stone burden, no perioperative complication, no residual stones needing auxiliary procedures, or depending on surgeon’s experience [31]. Shoma AM et al reported on 100 patients with upper tract calculi randomized to tubeless and standard PCNL group using closed envelops. The results of their study showed that tubeless PCNL might be unsuitable for the patients with CKD or a supracostal approach [36]. In our study, most patients with solitary kidney underwent Chinese MPCNL via the 11^th^ intercostal tract. Eleven patients had CKD stage 1, 15 had stage 2, 12 had stage 3, and 3 had stage 4. The associated coagulopathy in patients with CKD could increase the risk of hemorrhage, especially in the absence of nephrostomy tube. The nephrostomy tube plays an important role in draining the urine, establishing hemostasis, avoiding obstruction resulting from blood clots and residual fragments, and retaining the access tract for a staged procedure. Furthermore, a good drainage is very important to patients with solitary kidney underwent Chinese MPCNL via supracostal tract for avoiding secondary infection resulting from hematomas or urine extravasation, and hemothorax if blood clots obstruct the collecting system. Meanwhile, most patients in our study underwent Chinese MPCNL with guidance of ultrasonography, and the flexible ureteroscopy was not widely applied because of economic condition and medical insurance. The certain stone-free status needed to be evaluated postoperatively by imaging examinations. So the D–J stent use together with nephrostomy tube can be explained by little confidence in intraoperative assessment of residual stones and potential requirements for auxiliary procedures, including second-look PCNL, ESWL and flexible ureteroscopy. In our opinion, adequate drainage is very important for patients with solitary kidney in order to prevent urine extravasation, perinephric hematomas, obstruction resulting from the blood clots and residual fragments, and to preserve the renal function as far as possible. Because of the relative harsh healthcare environment of our country, doctors must critically evaluate the interventions that we perform with an eye toward improving their safety. Therefore, we are not very optimistic with the use of a single D–J stent or nephrostomy tube in patients with solitary kidney. Further studies are required to evaluate the safety of single-use D–J stent or nephrostomy tube in such patients.

Our study has some limitations as well. Firstly, this was a study about a small group of patients from a single institution. Secondly, because of the small working channel of the ureteroscope, the vision might not be clear, especially when patient had bleeding in operation. Thirdly, since this is a retrospective study, stone culture and associated metabolic study were not performed.

In conclusion, our clinical experience with Chinese MPCNL demonstrates that it is safe, feasible, and efficient for managing intrarenal calculi in solitary kidney with a satisfactory SFR and morbidity compared with standard PCNL. The renal function remained stable or even improved in the majority of patients underwent Chinese MPCNL with solitary kidney at both short-term and long-term follow-up.
